# Strategic Educational Expansion of Trauma Simulation Initiative via a Plan-Do-Study-Act Ramp

**DOI:** 10.5811/westjem.2022.12.57735

**Published:** 2023-01-11

**Authors:** Alexander Meshel, Laura Iavicoli, Barbara Dilos, George Agriantonis, Stuart Kessler, Phillip Fairweather, Devorah Nazarian, Daniel Lugassy, Suzanne Bentley

**Affiliations:** * The Mount Sinai Hospital, Department of Anesthesiology, Perioperative and Pain Medicine, New York, New York; † NYC H+H/Elmhurst, Elmhurst, New York; ‡ Icahn School of Medicine at Mount Sinai, NYC H+H/Elmhurst, Department of Emergency Medicine, New York, New York; § NYC H+H/Elmhurst, Department of Anesthesiology, Elmhurst, New York; ¶ NYC H+H/Elmhurst, Department of Emergency Medicine, Elmhurst, New York; || Icahn School of Medicine at Mount Sinai, Department of Anesthesiology, Perioperative and Pain Medicine, New York, New York; # Icahn School of Medicine at Mount Sinai, NYC H+H/Elmhurst, Department of Surgery, New York, New York; ** NYC H+H/Elmhurst, Simulation Center, NYC H+H/Elmhurst, Elmhurst, New York

## BACKGROUND

Trauma represents a major public health threat and is the leading cause of death for individuals 45 and older and the fourth leading cause of death in all ages.^1^ Trauma teams consist of a single team leader with a multidisciplinary group of people. Literature shows that having a designated leader improves team performance and that hands-off leaders with a directive style of leadership are more effective.^2^,^3^ However, despite consensus on the importance of leadership, clinical team leadership is often only a small component of broader teamwork-focused training.^4^ Simulation-based training is increasingly recognized as a mechanism to develop effective leadership and bolster team performance, including for trauma teams.^4^–^6^

In situ simulation offers a higher impact approach to trauma team training, allowing participants to participate as if a “real” trauma had occurred and allowing for systems assessment and capture of systems vulnerabilities.^7^,^8^ It requires high levels of coordination and facilitation and should not be initiated without serious pre-work and planning.

At our Level 1 Trauma Center, NYC Health + Hospitals/Elmhurst, there are two levels (red and yellow) of trauma team activation based on specific criteria, with each garnering a different level of responding team composition and resources. A “red trauma” is the highest level activation for the most critical and severely injured patients. Upon activation, a complex, multiprofessional, multidisciplinary group responds to the emergency department (ED) to work swiftly together as one team caring for the trauma patient.

## OBJECTIVES

With the known complexity of the trauma team and evidence supporting team training using simulation, our overarching objective was to demonstrate the use of a Plan-Do-Study-Act (PDSA) ramp as a framework for creating increasingly complex simulations in a stepwise, iterative fashion that could subsequently be used as a model for other simulations. We endeavored to create and implement in situ “red” (high acuity) trauma simulations with deliberately escalating but controlled complexity. This was done such that with each successive “upgrade” on the PDSA ramp, more complexity was systematically introduced by progressively adding layers of additional team members and resources to ultimately develop a full-scale, in situ simulation of the entire red trauma team and assisting consultants. We would use what was found in the previous level to both improve the clinical environment or system (eg, if a latent safety threat [LST] or area of opportunity was identified) and to inform future simulation iterations. Additionally, the simulation series itself was conducted with learning objectives related to trauma team leadership, team roles, communication, and clinical care decision-making, as well as use for assessment of trauma system functioning.

## CURRICULAR DESIGN

We used a novel educational approach incorporating a PDSA ramp to develop a realistically feasible and implementable full-scale, in situ trauma simulation initiative. We used trauma team performance observation checklists and debriefing discussion points to evaluate the “success” of the current cycle and to iteratively expand the simulation through the next PDSA cycle. If the main learning objectives were fulfilled and no critical clinical issues were identified, we progressed up the PDSA ramp. If we observed major clinical issues or failure to meet objectives, the current simulation was repeated before continuing up the ramp. Multiple simulations were conducted during each cycle. This trauma simulation initiative was determined to be exempt by the Institutional Review Board of the Icahn School of Medicine at Mount Sinai.

Each cycle followed the PDSA sequence:

- Plan: Simulation was planned and tailored to participant group(s), guided by specific educational learning objectives relevant to the scenario type and participating team.
- Do: Simulation was conducted followed by facilitated debriefing. During simulation, the trauma team performance observation checklist was completed by observers and debriefing discussion points were recorded during the debriefings.
- Study: Team performance, debriefing outcomes, and fulfillment of learning objectives were assessed, as well as areas of strength, identified LSTs, and opportunities for improvement—both educationally and in the clinical system.
- Act: Main points were reinforced, and education was provided to the team, as needed, via closed-loop debriefing with email back to participants summarizing findings and resulting changes/suggestions from debriefing outcomes. High-yield discussion points and learner-driven insights not previously incorporated in the simulation and debriefings were iteratively added to future simulation cycles. Identified LSTs were escalated to leadership for mitigation.

All simulations of the PDSA ramp followed the general outlined PDSA steps and had their own specific learning objectives. Each cycle possessed its own unique component of escalating complexity, as shown in the Figure. The simulation itself would range from 5–30 minutes based on the complexity of the stage, followed by 30 minutes of debriefing.

For example, PDSA1 consisted of planning (PLAN) and conducting (DO) two parallel yellow trauma simulations with emergency physicians in one simulation and ED nurses in a separate simulation. The PDSA1 main learning objectives focused on initial evaluation and management of minor trauma. A performance checklist and debriefing points were reviewed within the context of the learning objectives (STUDY). Main points were then reinforced, and education was provided to the team via closed-loop debriefing detailing a summary of findings and resulting changes/suggestions (ACT). Satisfactory completion of objectives allowed for expansion of the simulation to PDSA2; the same scenario completed this cycle by the now interdisciplinary ED team. Next, the same team completed a red trauma simulation. Following cycles continued systematic layering of additional team members, with increasing complexity in the learning objectives, through PDSA7 involving the entire trauma team (eg, ED, surgery, anesthesiology, respiratory therapy, registrars, techs, police).

In PDSA8 cycles, the entire red trauma team responded along with specialists based on specific case permutations and learning objectives crafted to necessitate different specialists (eg, neurosurgery, orthopedics, obstetrics, pediatrics). Most objectives by PDSA8 focus heavily on systems assessment and capturing vulnerabilities and LSTs, as well as the overarching goal of analyzing and maximizing leadership and overall teamwork and communication.

## IMPACT/EFFECTIVENESS

A total of 45 trauma simulations over 24 months were conducted across the aforementioned levels of complexity. Each stage involved different teams and different groups of participants based on objective. Some participants completed multiple stages. No participant completed the same stage twice. Of the 64 participants who completed post-evaluations following PDSA 7/8 (the culmination phases of the ramp), 100% agreed or strongly agreed that this was an effective clinical teaching tool, and more than 95% strongly agreed or agreed this would impact their future clinical practice, improve teamwork, and improve communication.

These simulations identified knowledge gaps (such as protocols, role assignments, and escalation pathways), performance deficiencies, and areas of need for additional training. Analyses of previous iterations identified system stressors that could be addressed both in real time and through future ramp cycles. Many of the critical team skills were able to be taught and reinforced in a manageable and streamlined manner (ie, leadership skills easier with smaller team structure, working up to commanding a much larger team in later cycles).

Of note, we recognize that trauma systems are individualized across institutions and that these specific PDSA ramps reflect our local environment. The PDSA ramp framework, however, applies to any trauma team, and the PDSA ramp table in the Figure can be customized to any trauma team system (or other clinical team). Deliberate reflection on local trauma protocols (eg, levels of trauma activation and resulting inclusion of specialists) can help guide the progressive ramps. While there are limitations, including buy-in of stakeholders and barriers to implementation, this novel application of a PDSA ramp approach adapted from quality improvement to in situ trauma-simulation creation may serve as an educational strategy for crafting meaningful future interventions and initiatives.

## Figures and Tables

**Figure:**
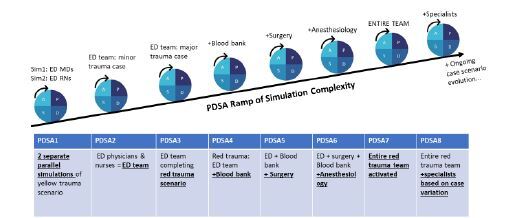
Plan-Do-Study-Act (PDSA) ramp with escalating complexity each cycle. *ED*, emergency department.
